# Increased Body Mass Index may lead to Hyperferritinemia Irrespective of Body Iron Stores

**DOI:** 10.12669/pjms.316.7724

**Published:** 2015

**Authors:** Faiza Alam, Abdul Shakoor Memon, Syeda Sadia Fatima

**Affiliations:** 1 Faiza Alam, Department of Biological and Biomedical Sciences, Aga Khan University, Stadium Road, Karachi - 74800, Pakistan; 2 Abdul Shakoor Memon, Department of Physiology, Basic Medical Sciences Institute, Jinnah Postgraduate Medical Centre, Karachi, Pakistan; 3 Syeda Sadia Fatima, Department of Biological and Biomedical Sciences, Aga Khan University, Stadium Road, Karachi - 74800, Pakistan

**Keywords:** Obesity, Inflammation, Ferritin, Iron, CRP

## Abstract

**Objective:**

Obesity causes subclinical inflammation which results in the secretion of various bioactive peptides that are key players in metabolic regulation of iron homeostasis. We sought to establish correlation of one such peptide (ferritin) with marker of subclinical inflammation (CRP) in various BMI.

**Methods:**

Total 150 subjects between the ages of 20-60 years were included in the cross-sectional study conducted at Basic Medical Sciences Institute, Jinnah Post Graduate Medical Centre, Karachi, Pakistan. Body Mass Index (BMI) was calculated by weight (kg) /height (m^2^). The given values were used as reference for Group A: normal weight (18.0-22.9 kg/m2), Group B: overweight (23.0-24.9 kg/m2), Group C: obese (>25.0 kg/m2) according to South Asian criteria. Serum Iron, Total Iron Binding Capacity, serum Transferrin Saturation, serum Ferritin and C-reactive protein were measured by commercially available kits. ANNOVA with Tukey’s minimum significant difference and Spearman Rho correlation were used considering p<0.05 significant.

**Results:**

The results identified an increased serum Ferritin and CRP in obese versus lean subjects (p < 0.001). BMI showed significantly positive correlation with serum CRP (r = 0.815; p-value < 0.01) and Ferritin (r = 0.584; p-value < 0.01). However, serum Iron levels and Transferrin saturation decreased in obese versus normal weight individuals (p < 0.001).

**Conclusion:**

This integrated new data reveals that individuals with high BMI had high levels of Serum Ferritin despite low levels of iron with high levels of C- reactive protein. This might be caused due to inflammatory conditions prevailing in the presence of increased adipose tissue.

## INTRODUCTION

Iron essentially a trace element; also possess properties of transition during mostbiological processes. Body iron is sourced from duodenal absorption, specifically in the heme form (Fe^+2^) and is released from both macrophages and liver.^1^ South Asian diet up to 90-95% comprises of non-heme iron of total daily iron requirement which is rather poorly absorbed by the gut.^2^ Researchers have shown that body iron is known to have a negative association with body mass index (BMI).^3^,^4^ Increased plasma volume and more significantly, inflammation induced by increased adiposity is suggested to be a link between iron status and adiposity.^5^ The C-reactive protein (CRP) is the first cytokine to be elevated in inflammatory conditions such as obesity. A strong relationship between obesity and CRP has been observed in all populations. This is because of the inflammatory changes in obese individuals. Concentration of CRP in serum decreases significantly after massive weight reduction.^6^ The pathophysiological mechanisms linking obesity toelevated levels of CRP are well recognized in obesity, the accumulation of free fatty acid intermediates activates pro-inflammatory serine kinase cascades.^7^

Among the many metabolic effects of obesity, higher BMI is also correlated with incongruity in iron parameters^8^ as this finding is attributed more to being physiological rather than due to dietary deficiency.^9^ Adipose tissue, the endocrine organ contributes to the development of inflammation process by secreting various pro-inflammatory cytokines and adipokines i.e. IL-6, TNF-α, leptin, C- reactive protein etc. Certain cytokines/chemokines influence food intake through direct effect of hypothalamus (Kershaw and Flier, 2004; Arslanet al., 2010). According to Zimmermann and Kohrle,^10^ chronic inflammation due to obesity results in low iron status. An inverse relationship has been found between physical activity and weight gain, and it has been suggested that physical inactivity could be another factor associated with decreased body iron in obesity in adults.^11^ It seems that increased body adipose tissue, particularly visceral depots, is associated with increased risk of iron deficiency which may be masked by high serum ferritin levels, presumably because the increase cytokines result in increased acute phase reactants synthesis resulting in increased macrophage sequestration and/or decreased intestinal iron absorption.^12^,^13^

Transferrin saturation and serum ferritin arethe preferential indicators for estimation of iron status.^14^ Ferritin expression is stimulated by several factors, such ascytokinereleased during inflammation and liver diseases. Inflammation in turn induces hepatic synthesis of acute phaseproteins. Obesity is one of the most common and prevalentconditionsthat promote this low grade inflammatory environment within the body.^15^

A relationship between iron status and inflammation utilizes C-reactive protein as an indicator of inflammation.^16^ Serum CRP concentration is utilized to support and authenticate the relationship between high ferritin levels in obesity with persistent low iron levels.^17^ However, no study has been carried out in South Asia up till now which establishes relationship between iron profile with South Asian BMI criteria.^18^,^19^ Keeping this in view, this proposed study aimed to elucidate whether obesity associated hypoferemia is correctly judged by serum levels of ferritin in obese individuals of South Asian and if it is correlated with subclinical inflammation. Thus we sought to estimate and correlate between low grade inflammatory marker (CRP) and iron marker (ferritin) in individuals with different BMI categories.

## METHODS

This cross-sectional study was conducted in Basic Medical Sciences Institute (BMSI), Jinnah Postgraduate Medical Centre, Karachi, Pakistan. The study was approved by the institutional ethical review board. Informed written consent was obtained from all study participants.

Total 380 healthy individuals between the ages of 20-60 years irrespective of gender were interviewed in detail regarding their medical, surgical and personal history to validate/confirm their suitability for inclusion in this study. Two hundred thirty individuals were excluded on the basis of conditions which influence the body iron stores such as pregnancy, alcoholism, hemoglobinopathies, diabetes mellitus, bleeding disorders, any acute illness during last one month, as well as iron deficiency as seen on complete blood picture (CP). Finally, 150 subjects were included in the present study. The subjects were divided into three groups with 50 individuals in each. Group A: normal weight (18.0-22.9 kg/m^2^), Group B: overweight (23.0-24.9 kg/m^2^), Group C: obese (>25.0 kg/m^2^)according to South Asian criteria of BMI.^20^,^21^

The weight and height of all the subjects were measured in kilograms and meters respectively, using a weight scale with a built-in Stadiometer (ZT -120 Health Scale, Nanjing EverichChina). Subjects were asked to stand in an erect posture wearing light clothing. Hip and waist circumference was measured by standard techniques.^22^ BMI was calculated using the following formula: (weight in kg / height in m^2^).^23^

About 6ml of blood was collected using all aseptic measures into EDTA vaccutainer (2 ml) and serum separator SSTs (4 ml). SST’s were centrifuged after complete clotting at 2000xG for 10 minutes. Clear serum was separated and stored in eppendorf at -80ºC until assayed. Hemoglobin (gm/dl) was measured on Sysmex (21 Cat No. KX21 manufactured by Kobe, Japan). Serum iron (mg/dl) and TIBC (mg/dl) were analyzed by enzymatic colorimetric method (kit Cat. No.61075 and kit Ref 61631 supplied by Bio Merieux S.A., France). Serum Ferritin (ŋg/dl) and CRP (mg/dl) were determined by Enzyme Linked Immuno-Sorbent Assay kit method (Cat No. BC- 1025 provided by BioCheck and Cat No. KAPDB 4360, manufactured by DIA Source, Immuno Assay S.A., Belgium), while transferrin saturation was calculated as 100x serum iron /TIBC.^24^

### Statistical Analysis

Data was analyzed using SPSS- version 19 (version 19; SPSS Inc., Chicago, IL, USA). Mean ± SD were calculated for quantitative variables. ANNOVA with Tukey’s minimum significant difference was used as a post hoc test, assuming homogeneity of variances. Spearman Rho correlation was applied to measure the relationship of obesity (as depicted by BMI)with ferritin, TIBC, iron and CRP and significance was considered at the level of p<0.05.

## RESULTS

The mean biophysical parameters of all three groups are shown in (Table-I). Mean weight was found significantly different between Group C when compared to Group A (p<0.001). The meanwaist and hip circumference among the three groups were also significantly different (p-value < 0.001), whereas no significant difference was observed in the waist to hip ratios among all three groups (p = 0.387). BMI in all three groups was significantly different (p<0.001).

**Table-I:** Biophysical and anthropometric variables among all groups.

Variables	Group A Control (n=50) Mean ±SD	Group B Overweight (n=50) Mean ±SD	Group C Obese (n=50) Mean ±SD
Age (years)	30.54±8.63	35.52±6.00	34.24±7.38
Weight (kg)	63.53±24.50	68.28±6.66	77.90±11.49*
Height (cm)	165.9± 8.53	168±7.84	163.18±9.19
Body Mass Index (kg/m^2^)	21.37±1.53	24.22±0.61*	29.29±4.1#
Waist Circumference (cm)	82.99±7.77	89.32±7.24*	97.12±10.45#
Hip Circumference (cm)	94.18±7.84	102.52±7.99*	107.72±11.01#
Waist Hip Ratio (W/H)	0.87 ±0.16	0.87 ±0.1	0.90 ±0.09

* significant as compared to controls, p<0.01

^ significant as compared to overweight, p<0.01

# significant as compared to controls and overweight p<0.01

**Fig.1:**
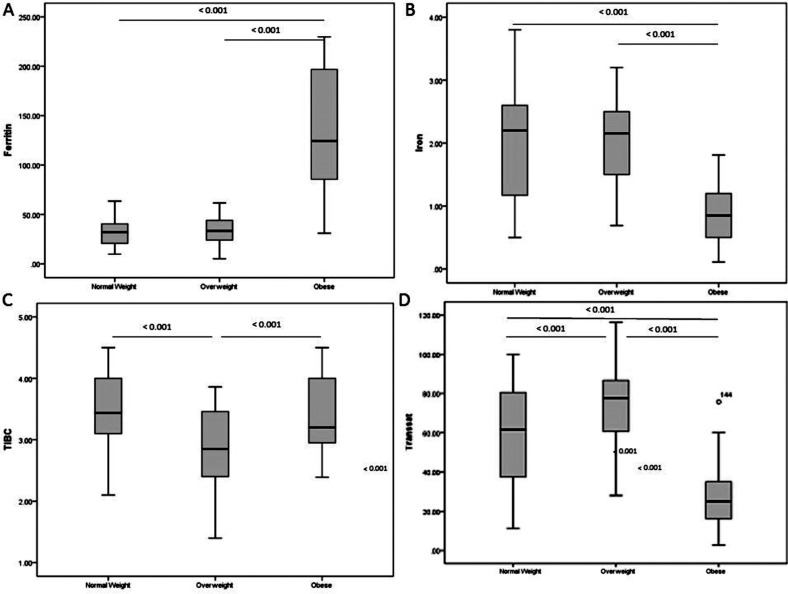
(A-D):Hemoglobin and iron parameters among all groups. Box plot shows the mean, 25^th^ and 75^th^ quartiles for Ferritin (a), Iron (b), TIBC (c) & Transferrin saturation (d)in three groups. X axis shows the study groups of normal weight (n= 50), overweight (n= 50) and obese (n= 50). Y axis shows the BMI obtained. Annovaposthoc test was applied for significant difference between Control vs. Overweight, Control vs. Obese and Overweight vs. Obese. P value<0.05 is considered significant.

The biochemical parameters are illustrated in (Table-II and Fig.1 A-D). No difference was observed in the meanhemoglobin values in all three groups. Serum ferritin levelswere high while serum iron were low in group Csubjects as compared to groups A and B (p < 0.001):TIBC was found to be significantly lower in group B (p < 0.001) as compared to group Awhile significantly higher in group Cas compared to group B (p < 0.001). Transferrin saturation percentage was high in group B as compared to group A (p = 0.001), and decreased in group Cas compared to the group A and group B (p < 0.001). The serum CRP levels depicted a rising trend from group A to C and showed significantly positive correlation with BMI (r = 0.815; p-value < 0.01) and Ferritin (r = 0.584; p-value < 0.01). Ferritin showed a positive correlation with increasing BMI (r = 0.614; p-value < 0.01) while negative correlation were observed for iron (r = -0.476; p-value < 0.01)and transferrin saturation percentage (r = -0.419; p-value < 0.01).(Table-III)

**Table-II:** Biochemical variables among all groups.

Variables	Group A Normal weight (n=50) Mean ±SD	Group B Overweight (n=50) Mean ±SD	Group C Obese (n=50) Mean ±SD
Hemoglobin (gm/dl)	13.04±2.68	12.7 ±1.66	13.62±1.77
Serum Ferritin (ŋg/dl)	31.62±13.23	34.23±12.45	136.15±59.3#
Serum Iron (mg/dl)	1.96±0.85	2.06±0.59	0.85±0.39#
Total Iron Binding Capacity (µg/dl)	3.46±0.61	2.84±0.61*	3.34±0.61^
Transferrin Saturation (trans sat%)	57.98±25.71	73.69 ±18.52*	26.63±13.90#
C- Reactive Protein (mg/dl)	2.22 ±1.11	3.83 ±1.12*	8.33 ±2.66#

* significant as compared to controls, p<0.01

^ significant as compared to overweight, p<0.01

# significant as compared to controls and overweight p<0.01

**Table-III:** Correlation of BMI with various study parameters.

Parameters	Spearman Rho correlation (r)	P value
Hemoglobin (g/dl)	0.080	0.18
Ferritin (ŋg/dl)	0.614	<0.01
Iron(mg/dl)	-0.476	<0.01
Total Iron Binding Capacity (mg/dl)	-0.038	0.25
Transferrin Saturation (Trans Sat%)	-0.419	<0.01
CRP(mg/dl)	0.815	<0.01
Correlation of Feritin with:
CRP	0.584	<0.01

Significant Correlation p<0.01, Where CRP: C- Reactive Protein;BMI: Body Mass Index.

## DISCUSSION

Obesity accounts for iron deficiency with or without anemia. This could be attributed to certain mechanisms associated to the pathogenesis of obesity, such as low grade inflammation.^25^ The trend noticed in this study was an increase in TIBC in overweight individuals when compared to normal weight, while iron concentration decreased. However, in obese individuals the TIBC levels tend to maintain near the normal values with persistent decreased iron levels (Table-II). These findings are contradictoryto what has been reported by Ghadiri et al,^26^ who described no change in TIBC with increase in BMI.

Studies have previously revealed lower levels of iron and transferrin saturation in obese as compared to the normal weight individuals and a negative correlation of transferrin saturation with BMI.^25^-^27^ Current study also supports this trend, suggesting that low iron levels could be a result of increased body iron demand, dilutional effect of increased plasma volume and most importantly, sequestration of iron through inflammatory-mediated mechanisms. Despite low iron levels no variations in hemoglobin levels was observed in study subjects. This observation could be related to the study’s exclusion criteria.

The surprising finding in this study was the elevated ferritin levels observed in the obese group along with a positive correlation with BMI. Various studiesconducted on obese metabolic syndrome patients versus obese non-metabolic syndrome patients reported higher levels and positive correlation of ferrit in with BMI between the groups, arguing against the presence of iron overload and advocating some level of inflammation responsible for high levels of ferritin (Table-II and Fig.1).^28^,^29^ In a recent study conducted by our group, we found elevated levels of serum ferritin, insulin, HOMA-IR and hs-CRP with low serum iron levels in Type II diabetics. We proposed that ferritin in combination with CRP may cause an undesirable inflammatory environment leading to insulin resistance.^30^

A study by Eftekhariet al.^31^ has demonstrated serum ferritin levels to be decreased in obesity. These discrepancies might be due to urbanization and lack of physical activity accompanied with junk food having less iron density. CRP is considered to be one of the earliest markers detected in inflammatory conditions. The study yielded high serum CRP levels in obese individuals. Our results suggest that obesity may cause activation of certain inflammatory mechanisms and increased cytokine secretion from adipose tissue, which in turn increases the hepatic secretion of CRP.^6^,^32^ In the obese group, with increasing BMI, iron concentration and transferrin saturation falls with ferritin and CRP concentration risefour folds. Thedecreased ironlevels in obese individuals might be potentiated by the inflammatory mechanisms, which entraps iron in the reticulo-endothelial system and activates the secretion of certain cytokines. Simultaneous increase in ferritin and CRPlevels in the presence of excessive fat content of the body may induceferritin to act as an acute phase reactant.

### Limitation of the study

The limitation of this study is its cross-sectional design. Comparative large population scale studies are not available to determine the baseline values of the iron parameters in our population. However, this study proposes a causal relationship between obesity, inflammation and low iron levels.

## CONCLUSION

Obese individuals show a unique picture of high ferritin and C- reactive protein, low serum iron and transferrin saturation. This may be an indication of fat cells playing a vital role in the production of acute phase reactants like ferritin which may not be the gold standard for the evaluation of iron status in obese individuals.
